# PlantLncBoost: key features for plant lncRNA identification and significant improvement in accuracy and generalization

**DOI:** 10.1111/nph.70211

**Published:** 2025-05-27

**Authors:** Xue‐Chan Tian, Shuai Nie, Douglas Domingues, Alexandre Rossi Paschoal, Li‐Bo Jiang, Jian‐Feng Mao

**Affiliations:** ^1^ School of Life Sciences and Medicine Shandong University of Technology Zibo Shandong 255000 China; ^2^ State Key Laboratory of Tree Genetics and Breeding, National Engineering Research Center of Tree Breeding and Ecological Restoration, National Engineering Laboratory for Tree Breeding, Key Laboratory of Genetics and Breeding in Forest Trees and Ornamental Plants, Ministry of Education, College of Biological Sciences and Technology Beijing Forestry University Beijing 100083 China; ^3^ Rice Research Institute, Guangdong Academy of Agricultural Sciences, Guangdong Key Laboratory of Rice Science and Technology, Guangdong Rice Engineering Laboratory, Key Laboratory of Genetics and Breeding of High Quality Rice in Southern China (Co‐construction by Ministry and Province) Ministry of Agriculture and Rural Affairs Guangzhou 510640 China; ^4^ Department of Genetics, “Luiz de Queiroz” College of Agriculture University of São Paulo 13418‐900 Piracicaba Sao Paulo Brazil; ^5^ Bioinformatics and Pattern Recognition Group (BIOINFO‐CP), Department of Computer Science Federal University of Technology – Paraná, UTFPR Campus Cornélio Procópio Cornélio Procópio 86300‐000 Brazil; ^6^ The Rosalind Franklin Institute OX110QX Didcot UK; ^7^ Department of Plant Physiology Umeå Plant Science Centre (UPSC), Umeå University Umeå 90187 Sweden

**Keywords:** feature selection, Fourier transform, gradient boosting algorithms, long noncoding RNAs (lncRNAs), model selection, ORF coverage

## Abstract

- Long noncoding RNAs (lncRNAs) are critical regulators of numerous biological processes in plants. Nevertheless, their identification is challenging due to the low sequence conservation across various species. Existing computational methods for lncRNA identification often face difficulties in generalizing across diverse plant species, highlighting the need for more robust and versatile identification models.
- Here, we present PlantLncBoost, a novel computational tool designed to improve the generalization in plant lncRNA identification. By integrating advanced gradient boosting algorithms with comprehensive feature selection, our approach achieves both high accuracy and generalizability. We conducted an extensive analysis of 1662 features and identified three key features – ORF coverage, complex Fourier average, and atomic Fourier amplitude – that effectively distinguish lncRNAs from mRNAs.
- We assessed the performance of PlantLncBoost using comprehensive datasets from 20 plant species. The model exhibited exceptional performance, with an accuracy of 96.63%, a sensitivity of 98.42%, and a specificity of 94.93%, significantly outperforming existing tools. Further analysis revealed that the features we selected effectively capture the differences between lncRNAs and mRNAs across a variety of plant species.
- PlantLncBoost represents a significant advancement in plant lncRNA identification. It is freely accessible on GitHub (https://github.com/xuechantian/PlantLncBoost) and has been integrated into a comprehensive analysis pipeline, Plant‐LncRNA‐pipeline v.2 (https://github.com/xuechantian/Plant‐LncRNA‐pipeline‐v2).

## Introduction

Long noncoding RNAs (lncRNAs) are key regulatory molecules in plants, influencing diverse biological processes, such as gene regulation, developmental pathways, and adaptive responses to environmental stresses (Wierzbicki *et al*., 2021; Yajnik *et al*., 2024). These molecules also interact with a variety of other noncoding RNAs, such as small RNAs (sRNAs), to modulate the complexity of gene regulatory networks and fine‐tune cellular functions (Traubenik *et al*., 2024). The identification and characterization of lncRNAs in plants have become increasingly important for advancing our understanding of plant biology and improving crop traits (Bhogireddy *et al*., 2021). However, their poor sequence conservation across species (Palos *et al*., 2023) poses a significant challenge for the generalization of machine learning models (Gudenas & Wang, 2018; Li & Liang, 2022). To address these challenges, strategic approaches including model selection, hyperparameter optimization, and feature selection following comprehensive feature extraction are critical for improving the accuracy of lncRNA identification (Bischl *et al*., 2023; Lv *et al*., 2023; Chen & Ghosh, 2024; Niu *et al*., 2024).

Model selection and feature selection are pivotal in enhancing the generalization of machine learning models, thereby improving their predictive performance (Negri *et al*., 2020). By carefully selecting the appropriate model, such as boosting models, such as Categorical Boosting (CatBoost), eXtreme Gradient Boosting (XGBoost), and Light Gradient Boosting Machine (LightGBM), which can mitigate the effects of multicollinearity and capture underlying patterns without overfitting (Li & Liang, 2022; Khalid *et al*., 2023), it is possible to achieve more reliable and accurate predictions. Hyperparameter tuning further refines this process, optimizing the model's performance on unseen data. Concurrently, feature selection plays a crucial role by eliminating irrelevant or redundant features, which not only simplifies the model and makes it more interpretable but also improves its ability to generalize from the training data to new, unseen data. Despite the robustness of these strategies for building machine learning models that are both accurate and generalizable, they are rarely implemented for plant lncRNA identification.

Robust and discriminative sequence features are essential for lncRNA identification. First, open reading frame (ORF)‐related features (Adjeroh *et al*., 2024), such as ORF length and coverage, leverage the fact that lncRNAs generally lack long ORFs, unlike mRNAs. Tools like Coding Potential Calculator (CPC) (Kong *et al*., 2007) and Coding Potential Assessment Tool (CPAT) (Wang *et al*., 2013) utilize ORF features to estimate coding potential. Second, nucleotide composition features, including guanine‐cytosine (GC) content, *k*‐mer frequencies, and Fickett score, are rooted in the statistical distribution of nucleotides within sequences, as seen in predictor of long non‐coding RNAs and messenger RNAs based on an improved k‐mer scheme (PLEK) (Li *et al*., 2014), RNAplonc (Negri *et al*., 2018), and FlExible Extraction of LncRNAs (FEELnc) (Wucher *et al*., 2017), where lncRNAs often exhibit distinct patterns from mRNAs. Third, sequence conservation is based on the assumption that functional noncoding RNAs will show evolutionary conservation across species, even without coding regions. This approach is implemented in tools like Phylogenetic Codon Substitution Frequency (PhyloCSF) (Lin *et al*., 2011) and Lncrna Linear Order cOnserved Motifs (LncLOOM) (Ross *et al*., 2021), which detect functional lncRNAs by comparing conservation patterns. Additionally, gene structure features are utilized in distinguishing lncRNAs from mRNAs, which is implemented in tools like LncRScan‐SVM (Sun *et al*., 2015). Furthermore, Plant Long Non‐Coding rna Prediction by Random fOrests (PLncPRO) (Singh *et al*., 2017) integrates a broad range of sequence features, such as ORF coverage and Blastx results, with a random forest model to improve lncRNA identification in plant genomes. LncMachine (Cagirici *et al*., 2021) employs a combination of sequence‐based features, including *k*‐mer frequencies, ORF‐related metrics, Fickett scores, and isoelectric point predictions, to distinguish lncRNAs from coding RNAs specifically in crop species.

In recent advances, mathematical descriptors have shown great potential for lncRNA identification. Among thousands of mathematical features of DNA/RNA sequences, Fourier transform features extract periodic signals and frequency‐domain information from sequences (Messaoudi *et al*., 2014), while entropy‐based features (e.g. Shannon and Tsallis entropy) quantify sequence complexity and randomness as implemented in an *ab initio* lncRNA identification and functional annotation tool based on deep learning (LncADeep) (Yang *et al*., 2018). The extensive array of DNA/RNA features, such as those introduced in MathFeature, facilitates a more profound comprehension of sequence properties by capturing intricate patterns that extend beyond traditional biological features.

In this study, we undertook a comprehensive approach to model selection, hyperparameter optimization, and feature selection, aimed at advancing the predictive accuracy of plant lncRNA identification. Following feature extraction, we specifically focused on three key features that were selected from 1433 conventional and 219 novel mathematical descriptors. Finally, we developed PlantLncBoost, a computational model designed to address the challenges of generalization and accuracy in plant lncRNA identification. By leveraging both traditional and innovative mathematical descriptors, PlantLncBoost enhances prediction accuracy and offers deeper biological insights.

## Materials and Methods

### Training and test data collection

For the construction of our classification model, we utilized lncRNA and mRNA datasets from nine diverse angiosperm species (Table 1). The selected species were *Amborella trichopoda* Baill., *Arabidopsis thaliana* (L.) Heynh, *Brachypodium distachyon* (L.) P. Beauv., *Citrus sinensis* (L.) Osbeck, *Cucumis sativus* L., *Glycine max* (L.) Merr., *Oryza sativa* L., *Populus trichocarpa* Torr. & A. Gray, and *Ricinus communis* L. Redundant sequences with over 80% sequence identity were removed using CD‐HIT‐EST (Li & Godzik, 2006). Additionally, sequences containing ambiguous nucleotides (represented as ‘N’) were discarded to reduce noise and uncertainty. A total of 24 152 lncRNA sequences were obtained from GreeNC (Di Marsico *et al*., 2022), a database employing stringent criteria for high‐quality plant lncRNA selection. An equal number of mRNA protein‐coding sequences were obtained from Phytozome v.13 (https://phytozome.jgi.doe.gov/), in order to guarantee a balanced training set for our supervised learning model.

**Table 1:** The lncRNA and mRNA data used for model training.

Species	lncRNA		mRNA	
	GreeNC	Used	Phytozome	Used
*Amborella trichopoda*	5698	4556	26 846	4556
*Arabidopsis thaliana*	3008	1803	35 386	1803
*Brachypodium distachyon*	5584	4877	46 147	4877
*Citrus sinensis*	2562	2215	27 775	2215
*Cucumis sativus*	3987	1803	46 147	1803
*Glycine max*	2562	1804	30 364	1804
*Oryza sativa*	1929	1803	88 647	1803
*Populus trichocarpa*	6689	1804	39 068	1804
*Ricinus communis*	4198	3487	31 221	3487

To evaluate the effectiveness of our model, we used a comprehensive test set consisting of lncRNAs from 20 plant species, including *Amborella trichopoda*, *Ananas comosus* (L.) Merr., *Arabidopsis thaliana*, *Brachypodium distachyon*, *Cucumis sativus*, *Glycine max*, *Manihot esculenta* Crantz, *Medicago truncatula* Gaertn., *Musa acuminata* Colla, *Oryza sativa*, *Populus trichocarpa*, *Solanum lycopersicum* L., *Sorghum bicolor* L., *Vitis vinifera* L., *Zea mays* L., *Chlamydomonas reinhardtii* P.A. Dang., *Coccomyxa subellipsoidea*, *Micromonas pusilla* (Butcher) Manton & Parke, *Volvox carteri* F. Stein, and *Physcomitrella patens* (Hedw.) Bruch & Schimp., which were ever used previously (Tian *et al*., 2024) (Supporting Information Table S1). This diverse array of species was used in order to verify its general applicability in plant lncRNA classification. To compile a high‐confidence set of experimentally validated lncRNAs, data were integrated from two databases: experimentally validated lncRNAs (EVLncRNAs) (Zhou *et al*., 2018; Zhou *et al*., 2024); v.1.0 and updated v.3.0) and plant long non‐coding RNA database (PlncDB) (Jin *et al*., 2020), which aggregates highly reliable lncRNAs previously curated from EVLncRNAs v.1.0 (Zhou *et al*., 2018). Initially, overlaps between the two databases were identified, resulting in the removal of 55 redundant transcripts. Consequently, a total of 358 unique, experimentally validated lncRNAs were retained for further analysis, distributed across 20 plant species (Table S2), with lncRNAs from 12 species not included in both training and test sets, that is *Brassica napus*, *Brassica rapa*, *Daucus carota*, *Ganoderma lucidum*, *Gossypium barbadense*, *Gossypium hirsutum*, *Malus domestica*, *Panax ginseng*, *Raphanus sativus*, *Salvia miltiorrhiza*, *Triticum aestivum*, and *Vigna radiata*.

### Model selection

To identify novel predictive features for plant lncRNAs and efficiently classify these sequences, we evaluated three gradient boosting algorithms known for their effectiveness in handling complex biological datasets: CatBoost (Dorogush *et al*., 2018), XGBoost (Chen & Guestrin, 2016), and LightGBM (Ke *et al*., 2017). CatBoost incorporates ordered categorical features and reduces overfitting through advanced target‐based encoding, while XGBoost is known for robust performance through optimized gradient boosting and regularization techniques. LightGBM, in turn, offers superior computational efficiency by employing histogram‐based algorithms and leaf‐wise tree growth (Ke *et al*., 2017). Given the high dimensionality and complexity of datasets, our analysis placed particular emphasis on assessing computational efficiency and scalability, which are crucial for large‐scale genomic analyses. All analyses were executed on a server equipped with AMD EPYC 7H12 processors. This server features a dual‐socket configuration, with each socket containing 64 cores and each core supporting 2 threads.

### Feature extraction

MathFeature (Bonidia *et al*., 2021) was utilized to extract features for the construction of the lncRNA prediction model. This tool can extract multiple sequence characteristics, including ORF length, coverage, *k*‐mer frequencies, and a variety of novel mathematical features. By converting biological sequences (DNA, RNA, and proteins) into numerical information, MathFeature facilitates a comprehensive analysis of nucleotide sequences from both mathematical and statistical perspectives. In this study, a total of 1662 features were extracted, encompassing basic sequence characteristics (e.g. ORF coverage, *k*‐mer frequencies, and Fickett score), numerical mappings, Fourier transforms, entropy measures, and complex network features.

### Identification of optimal feature subset

Feature selection is essential for building accurate plant lncRNA prediction models. Here, we systematically evaluated six distinct methods for feature selection: Pearson correlation coefficient, ANOVA correlation coefficient, mutual information, recursive feature elimination (RFE), random forest importance (RFI), and variance threshold (VT). The VT method was applied to eliminate features with a variance below 0.01, thereby removing near‐constant features. For other selection methods, we constructed and compared models based on the top 10 features identified. These selected feature sets were then utilized as inputs for model optimization. Bayesian hyperparameter optimization (Zhang *et al*., 2020) was applied using the CatBoost algorithm to fine‐tune the parameters for each feature set.

We employed a 10‐fold cross‐validation strategy to evaluate and compare the performance of various feature selection methods. The training dataset was randomly divided into 10 equal subsets. In each iteration, nine subsets were used for model training, while the remaining subset served as the test set for evaluation. This process was iterated 10 times, ensuring that each subset was used once as the test set. The final evaluation metrics for each model were determined by calculating the mean performance metrics across all iterations.

### Model construction and implementation

**Fig. 1:**
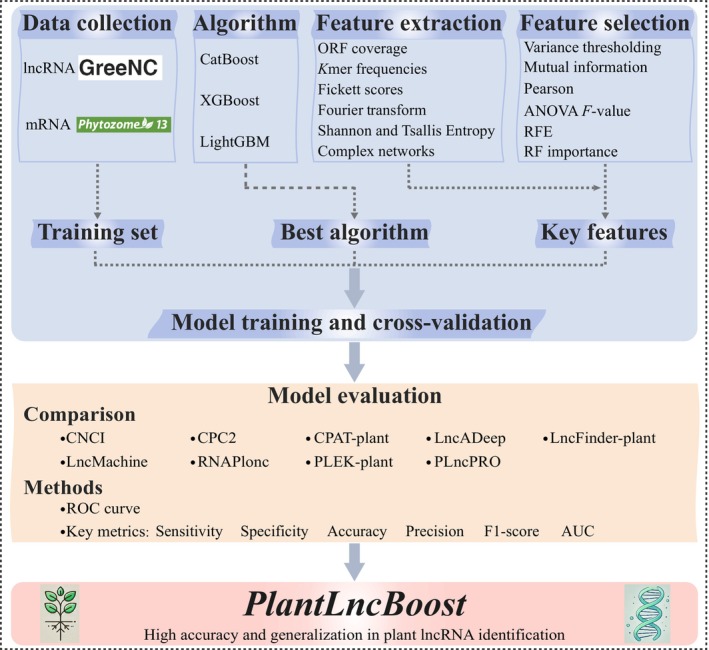
Workflow of PlantLncBoost development. ORF, open reading frame; AUC, area under the curve; RFE, recursive feature elimination. RF, random forest; CNCI, Coding‐Non‐Coding Index; CPC, Coding Potential Calculator; CPAT, Coding Potential Assessment Tool; PLEK, predictor of long noncoding RNAs and messenger RNAs based on an improved *k*‐mer scheme; ROC, Receiver operating characteristic; CatBoost, Categorical Boosting; lncRNA, Long noncoding RNA; P; LncPRO, plant long noncoding RNA prediction by random forests; LncFinder, an integrated platform for long non‐coding RNA identification.

Building upon the algorithm evaluation, feature extraction and selection, and hyperparameter optimization procedures described above, we developed an innovative prediction model for plant lncRNAs, named PlantLncBoost. The complete model construction process is shown in Fig. 1. PlantLncBoost is available in the GitHub repository, https://github.com/xuechantian/PlantLncBoost.

## Results

### Comprehensive sequence and feature collection

The training set consisted of 24 152 lncRNA and 24 152 mRNA sequences from nine species (Table 1). The test set included a collection of 144 268 lncRNA and 144 268 mRNA sequences from 20 species, among which 13 species were not included in training (Table S1) (Tian *et al*., 2024). To identify critical features for training a robust lncRNA model, we extracted a set of 1662 features (Table S3) from our training dataset. This set includes both conventional sequence‐based metrics – such as ORF coverage, *k*‐mer frequencies, and Fickett scores – and novel mathematical features designed to capture intricate sequence patterns (Table S3). In particular, 1433 of these features are fundamental sequence descriptors, while 133 result from numerical sequence mapping and Fourier transforms. We also included 78 complex network features and 19 features derived from Shannon and Tsallis entropy.

### Gradient boosting algorithms: model selection

In a comparative analysis of three gradient boosting algorithms (CatBoost, XGBoost, and LightGBM), we utilized fivefold cross‐validation on a the training dataset. CatBoost consistently outperformed the other algorithms, demonstrating superior performance and faster model construction times compared to XGBoost and LightGBM. Specifically, CatBoost achieved the highest accuracy of 93.92%, a sensitivity of 99.83%, and an F1‐score of 94.30%, surpassing both XGBoost and LightGBM (Table S4).

During hyperparameter optimization, CatBoost proved highly efficient, requiring only 14.45 min to evaluate parameter combinations with fivefold cross‐validation. By contrast, XGBoost required 164.18 min, and LightGBM required 55.67 min to complete the same task (Table S5). This result underscores the superior performance of CatBoost in model tuning compared to the other algorithms. Moreover, constructing the final model with optimized hyperparameters in CatBoost takes *c*. 19.41 min, while XGBoost and LightGBM require 53.89 and 25.58 min, respectively (Table S5). CatBoost also excelled in lncRNA prediction, producing results in under 10 s (Table S5). Consequently, we identified CatBoost as the best gradient boosting algorithm for plant lncRNA classification.

### Feature selection methods

**Fig. 2:**
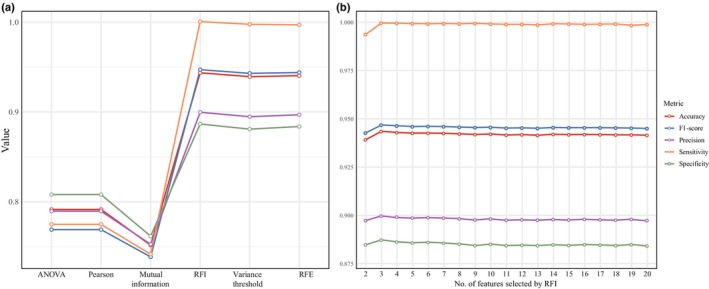
Fivefold cross‐validation evaluation of features selected with different feature selection methods. (a) The comparative assessment of different feature selection methods, including ANOVA, Pearson correlation coefficient, mutual information, random forest importance (RFI), variance threshold, and recursive feature elimination (RFE). (b) The comparative assessment based on RFI method and different numbers of features.

The optimal hyperparameter configurations for each feature selection method are detailed in Table S6. Fivefold cross‐validation results demonstrated that the model employing RFI feature selection outperformed others across key evaluation metrics (Fig. 2a). The RFI‐based model achieved an accuracy of 94.21%, an F1‐score of 94.56%, a precision of 89.82%, a sensitivity of 99.91%, and a specificity of 88.51% (Table S7).

Models based on VT and RFE methods performed second best to those of RFI‐based method. By contrast, filter‐based methods such as analysis of variance, Pearson correlation coefficient, and mutual information demonstrated relatively poorer performance, with accuracies ranging between 75% and 79% and F1‐scores *c*. 77% (Fig. 2a; Table S7). These findings indicate that the feature selection strategy utilizing RFI is the optimal choice for subsequent analyses.

### Selection of key features

To determine the optimal number of features for model construction using the RFI method, we evaluated models incorporating the top 1 to 20 ranked features identified by RFI. Hyperparameter optimization was conducted for each model to ascertain the best parameter combinations (Table S8). The fivefold cross‐validation results indicated that the highest performance metrics were achieved with the top three features, as represented in the RFI‐3 model (Fig. 2b; Table S9). The RFI‐3 model exhibited superior performance with an accuracy of 94.35%, an F1‐score of 94.68%, a precision of 89.99%, a sensitivity of 99.96%, and a specificity of 88.73% (Table S9). Performance metrics began to decline when the model included more than three features (Fig. 2b).

The RFI‐3 model incorporated three key predictive features: ORF coverage, complex Fourier average, and atomic Fourier amplitude. ORF coverage, which represents the proportion of ORFs in a sequence, is critical for distinguishing coding sequences from noncoding ones (Wang *et al*., 2013). Complex Fourier Average and Atomic Fourier amplitude are novel mathematical features derived from Fourier transformation. To digitize RNA sequences for Fourier transformation, seven numerical mapping techniques were employed, including binary, *Z*‐curve, real, integer, Electron‐Ion Interaction Potential (EIIP), complex number, and atomic number encodings. Complex Fourier average and atomic Fourier amplitude were specifically derived from the complex number and atomic number encoding methods, respectively. These features may capture significant sequence or structural information pertinent to plant lncRNAs.

### Exploration of key features

The further analysis across three model plant species (*Arabidopsis thaliana*, *Oryza sativa*, and *Populus trichocarpa*) demonstrated that ORF coverage, complex Fourier average, and atomic Fourier amplitude are robust features for distinguishing lncRNAs from mRNAs (Fig. S1). ORF coverage showed a clear separation between lncRNAs and mRNAs in all three species. For instance, in *A. thaliana*, lncRNAs peaked at lower ORF coverage values (*c*. 0.2), while mRNAs peaked at higher values (*c*. 0.7) (Fig. S1). This pattern was also observed in *O. sativa* and *P. trichocarpa*, with slight variations in peak positions, highlighting the universal applicability of this feature across diverse species. Similarly, complex Fourier average and atomic Fourier amplitude exhibited significant classification potential (Fig. S1). In all three species, lncRNAs consistently peaked at lower values, whereas mRNAs had a broader distribution skewed toward higher values.

Additionally, we evaluated traditional sequence‐based features, including *k*‐mer frequencies and Fickett scores, for their effectiveness in distinguishing lncRNAs. Both features exhibited limited discriminatory power across all species (Figs S2, S3). Specifically, the *k*‐mer distributions showed substantial overlap between lncRNAs and mRNAs, while Fickett scores, despite showing slight separation, still presented considerable overlap.

**Fig. 3:**
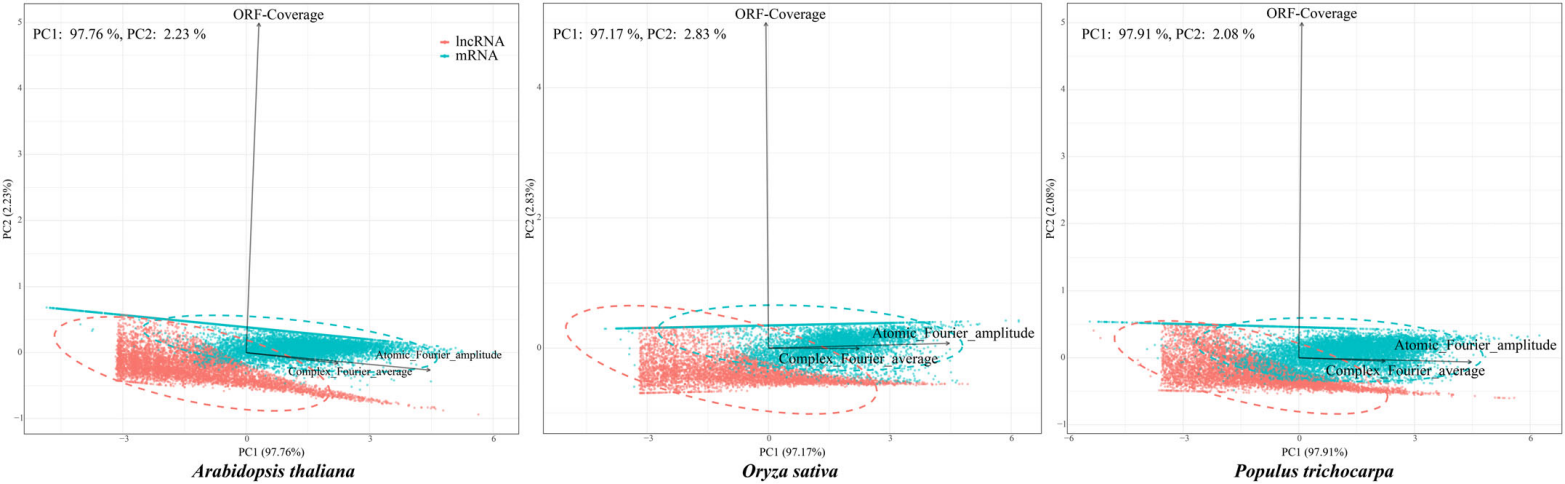
Principal component analysis of lncRNA and mRNA based on three key features across three model species. LncRNA, long noncoding RNA; ORF, open reading frame; PC1, first principal component; PC2, second principal component.

Principal component analysis (PCA) revealed a distinct separation between lncRNAs and mRNAs based on three key features (Fig. 3). The two fourier‐based features complex Fourier average and atomic Fourier amplitude, were primarily responsible for the separation along the first principal component, which accounted for up to 97% of the variance across *A. thaliana*, *O. sativa*, and *P. trichocarpa* (Fig. 3). This suggests that these features capture essential differences between lncRNAs and mRNAs. Meanwhile, ORF coverage contributed to the variance along the second principal component, providing further discriminatory power (Fig. 3).

These results underscore the effectiveness of our feature selection, emphasizing the potential to enhance lncRNA prediction models by prioritizing Fourier‐based features for primary classification. Additionally, a minor overlap between lncRNAs and mRNAs likely indicates RNAs with intermediate traits or shared features (Fig. 3). This overlap may also result from annotation discrepancies or other factors, complicating the clear distinction between lncRNAs and mRNAs.

### Modeling with key features

Utilizing the CatBoost algorithm and three key features, we developed an enhanced plant lncRNA prediction model, PlantLncBoost. Tenfold cross‐validation results demonstrated that PlantLncBoost outperforms the leading models, LncFinder‐plant and CPAT‐plant (Fig. S4). Specifically, PlantLncBoost achieved an accuracy of 94.35%, an F1‐score of 94.68%, a precision of 89.99%, a sensitivity of 99.96%, and a specificity of 88.73% (Fig. S4). These metrics collectively highlight the robust predictive power of PlantLncBoost in differentiating plant lncRNAs from mRNAs.

### Benchmarking of multiple models

**Fig. 4:**
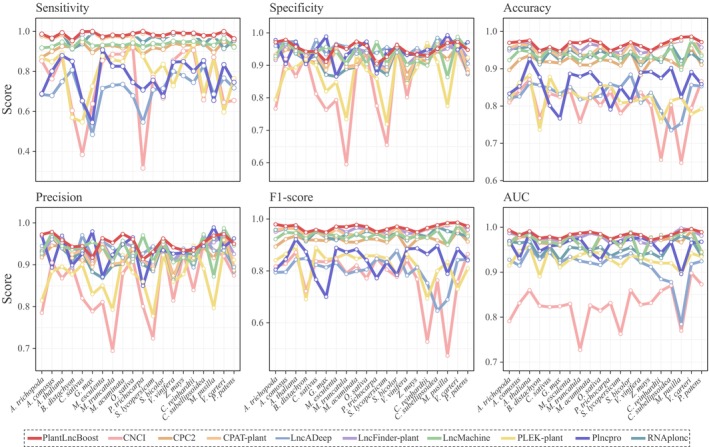
Performance evaluation of 10 lncRNA identification tools on 20 plant datasets. From left to right, the panels present Sensitivity (proportion of true positives correctly identified), Specificity (proportion of true negatives correctly identified), Accuracy (overall correctness of predictions), Precision (proportion of positive identifications that were correct), F1‐score (harmonic mean of precision and sensitivity), and AUC (area under the ROC curve, measuring overall discriminative ability).The x‐axis in each panel represents the 20 different plant species tested. PlantLncBoost consistently shows superior performance across most species and evaluation metrics. AUC, area under the curve; CNCI, Coding‐Non‐Coding Index; CPC, Coding Potential Calculator; CPAT, Coding Potential Assessment Tool; lncRNA, long noncoding RNA; PLEK, predictor of long noncoding RNAs and messenger.

**Fig. 5:**
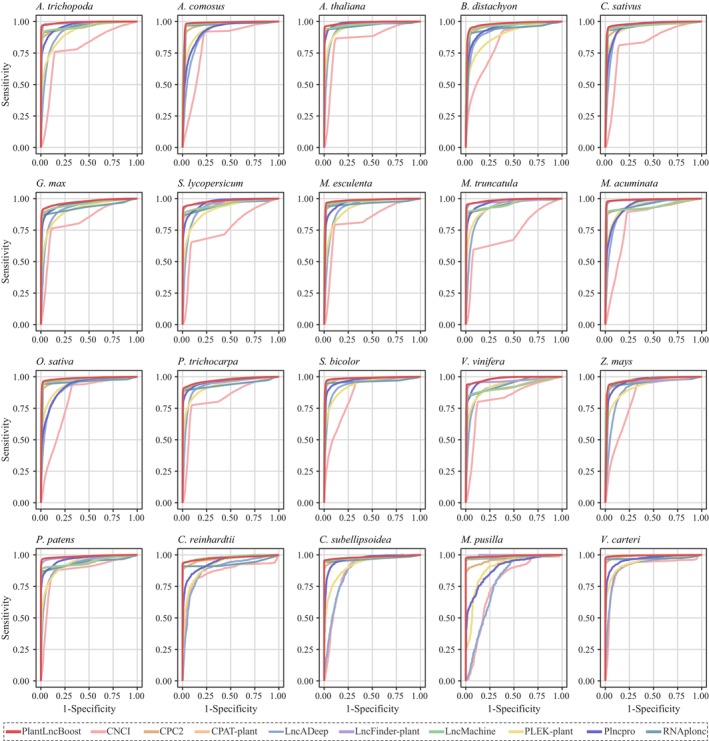
ROC curve of 10 lncRNA identification methods on 20 plant datasets. Each panel displays the performance of different lncRNA identification methods (PlantLncBoost, CNCI, CPC2, CPAT‐plant, LncADeep, LncFinder‐plant, LncMachine, PLEK‐plant, PlncPro, and RNAplonc) on a separate plant species. The species analyzed include *Amborella trichopoda, Ananas comosus, Arabidopsis thaliana, Brachypodium distachyon, Cucumis sativus, Glycine max, Solanum lycopersicum, Manihot esculenta, Medicago truncatula, Musa acuminata, Oryza sativa, Populus trichocarpa, Sorghum bicolor, Vitis vinifera, Zea mays, Physcomitrella patens, Chlamydomonas reinhardtii, Coccomyxa subellipsoidea, Micromonas pusilla*, and *Volvox carteri*. Higher curves toward the upper‐left corner indicate better performance. CNCI, Coding‐Non‐Coding Index; CPC, Coding Potential Calculator; CPAT, Coding Potential Assessment Tool; lncRNA, long noncoding RNA; PLEK, predictor of long noncoding RNAs and messenger.

We benchmarked our new model, PlantLncBoost, against nine established lncRNA prediction models: LncFinder‐plant, CPAT‐plant, RNAplonc, PLncPRO, CPC2, LncDeep, LncMachine, PLEK‐plant, and CNCI (Table S10). The evaluation used test datasets from 20 diverse plant species of a broad range of plant lineages, that is Spermatophytes, Bryophyte, and Archaeplastida. The results indicated that PlantLncBoost outperformed all other models across key metrics, achieving the highest values in sensitivity (98.42%), specificity (94.93%), accuracy (96.63%), precision (95.14%), area under the curve (AUC) (98.35%), and F1‐score (96.74%) (Tables 2, S10, S11). Remarkably, the model demonstrated near 100% sensitivity in most species while maintaining specificity and precision above 90% consistently (Fig. 4; Table S10). This enhancement in sensitivity did not compromise specificity, highlighting the robustness of the model and its ability to balance critical performance metrics for accurate plant lncRNA prediction. The receiver operating characteristic (ROC) curve for PlantLncBoost was notably closer to the top‐left corner, underscoring its superior predictive capability across the majority of tested species (Fig. 5).

**Table 2:** Overall performance of 10 lncRNA identification methods on datasets from 20 plant species.

Models	Sensitivity (%)	Specificity (%)	Accuracy (%)	Precision (%)	F1‐score (%)	AUC (%)
**PlantLncBoost**	**98.42**	**94.93**	**96.63**	**95.14**	**96.74**	**98.35**
LncFinder‐plant	98.18	93.28	95.73	93.64	95.84	97.88
CPAT‐plant	97.86	92.44	95.15	92.91	95.30	97.08
RNAplonc	96.71	91.63	93.68	92.06	94.26	95.22
LncMachine	93.68	93.58	93.63	93.68	93.65	96.99
CPC2	91.98	91.88	91.93	91.94	91.93	96.63
PLncPRO	77.26	94.36	85.81	93.47	84.19	95.82
LncADeep	71.07	94.79	82.93	93.32	80.37	91.60
PLEK‐plant	79.61	87.05	82.12	87.15	82.44	92.92
CNCI	74.59	85.58	80.08	85.49	77.86	82.70

AUC, area under the curve; LncADeep, an *ab initio* lncRNA identification and functional annotation tool based on deep learning. Values in bold represent the highest scores in each column.

LncFinder‐plant and CPAT‐plant (Tian *et al*., 2024) followed closely behind PlantLncBoost in overall performance (Figs 4, 5; Table 2). Notably, RNAplonc also demonstrated commendable performance with a high sensitivity of 96.71%, an accuracy of 93.68%, and an AUC of 95.22%, positioning it as the fourth best‐performing tool in our comparative analysis. LncMachine showed comparable accuracy (93.63%) to RNAplonc but with lower sensitivity (93.68%). By contrast, PLncPRO demonstrated relatively lower sensitivity, accuracy, and F1‐score, though it still showed competitive specificity and precision (Table 2). Meanwhile, CPC2, LncDeep, PLEK‐plant, and CNCI exhibited significantly lower accuracy and F1‐scores, ranging between 80% and 90%, reflecting a marked underperformance compared to the other tools (Table 2).

### Benchmarking with experimentally validated plant lncRNAs

In this benchmarking with experimentally validated lncRNAs (Table S2), PlantLncBoost and LncFinder‐plant both achieved the highest detection rate, identifying 357 of 358 lncRNAs (99.72%). CPAT‐plant followed closely with 355 lncRNAs (99.16%), while CPC2, LncMachine, PLncPRO, and RNAplonc each recognized 353 (98.60%). By contrast, CNCI captured 333 transcripts (93.02%), LncADeep identified 321 (89.66%), and PLEK‐plant had the lowest detection rate at 311 (86.87%).

Notably, the single lncRNA (GenBank: KC549675.1, from *Triticum aestivum* and it was designated as TalncRNA18) (Zhang *et al*., 2013) that escaped detection by PlantLncBoost was consistently classified as a protein‐coding RNA by all other tools. Revisiting its initial characterization revealed critical methodological limitations: the original classification as a lncRNA depended exclusively on ORF detection using the legacy NCBI ORF Finder (Zhang *et al*., 2013), which failed to identify any significant ORF. However, modern multi‐feature tools (LncFinder‐plant, CPAT‐plant, lncMachine, CNCI, CPC2, etc.) all predicted the ORF spanning > 100 amino acids, with coding potential scores surpassing empirical thresholds. Further analysis using the updated NCBI ORF Finder identified 12 putative ORFs, with the longest ORF encoding a 387‐amino‐acid polypeptide, exhibiting sequence homology to E3 ubiquitin‐protein ligase UPL1‐like gene.

In summary, benchmarking with experimentally validated lncRNAs suggested PlantLncBoost, LncFinder‐plant, and CPAT‐plant as the three most effective models for predicting plant lncRNAs. The plant lncRNA identification model developed in this research, PlantLncBoost, is freely accessible to the scientific community at https://github.com/xuechantian/PlantLncBoost.git. Additionally, this model has been integrated into a comprehensive lncRNA analysis pipeline, Plant‐LncRNA‐pipeline v.2, available at https://github.com/xuechantian/Plant‐LncRNA‐pipeline‐v2. This pipeline encompasses a series of processes, including raw data filtering, transcriptome alignment and assembly, lncRNA prediction using PlantLncBoost, CPAT‐plant, and LncFinder‐plant, lncRNA classification, and origin analysis. Additionally, if users wish to use more tools, RNAplonc is worth considering due to its commendable performance in our evaluation.

## Discussion

### Challenge of weak generalization in lncRNA identification

The core challenge addressed in this study is the weak generalization of existing models for plant lncRNA identification, which primarily results from poor sequence conservation among lncRNAs across diverse plant species (Budak *et al*., 2020). Traditional models such as LncFinder and CPAT, which were originally designed for nonplant species, exhibit limitations when generalized to plants due to the reliance on features and classification strategies that are insufficient for capturing the specific properties of plant lncRNAs (Tian *et al*., 2024). To overcome these limitations, PlantLncBoost integrates advanced gradient boosting algorithms and novel mathematical features, providing a more versatile and generalizable approach to lncRNA identification in plants. The application of CatBoost following model selection, combined with comprehensive feature selection, allows PlantLncBoost to capture essential differences between lncRNAs and mRNAs that are consistent across a wide variety of plant species, as it was demonstrated in benchmarkings with lncRNAs from diverse plant lineages, especially those that are experimentally validated.

The weak generalization of previous lncRNA identification models has been a significant bottleneck in research involving plant lncRNAs. The lack of sequence conservation among plant lncRNAs makes it difficult for conventional models, which often depend on sequence similarity or some secondary structure features, to distinguish between coding and noncoding RNAs effectively. Our approach with PlantLncBoost addresses this by selecting features that do not rely solely on sequence similarity but instead focus on mathematical properties and signal characteristics of nucleotide sequences, leading to a substantial improvement in cross‐species generalization.

### Comparison of feature selection methods

Feature selection is pivotal in constructing high‐performance predictive models (Bonidia *et al*., 2020), especially for increasing generalization in distinguishing plant lncRNAs from mRNAs. Effective feature selection not only eliminates redundant and irrelevant information but also enhances prediction accuracy and generalization ability (Guyon *et al*., 2008; Storcheus *et al*., 2015; Zhou *et al*., 2021). In this study, we systematically compared various feature selection methods, including RFI, RFE, VT, and several filter‐based approaches, such as Pearson correlation coefficient, ANOVA, and mutual information.

Our cross‐validation results demonstrated that the RFI‐based feature selection strategy outperformed the others across key evaluation metrics. This superior performance is likely due to the random forest algorithm's ability to automatically learn and exploit high‐order interactions and nonlinear patterns among features, as well as its strong resistance to noise and overfitting (Akhiat *et al*., 2021). RFE also showed competitive performance, ranking closely behind RFI. By recursively eliminating the least important features based on model performance, RFE effectively captures complex feature interactions, leading to improved model accuracy. However, RFE is computationally intensive, especially when dealing with large feature sets, as it requires retraining the model multiple times to evaluate the importance of each feature subset. This significant time cost makes RFE more suitable for small sample datasets (Chen & Jeong, 2007). In datasets with high dimensionality, such as biological sequence data with numerous features, the computational cost of RFE becomes prohibitive, limiting its practicality for large‐scale analyses. By contrast, filter‐based methods like Pearson correlation coefficient, ANOVA, and mutual information exhibited a clear lack of competitiveness. These univariate methods fail to effectively capture high‐order interactions and complex correlation patterns among features (Saeys *et al*., 2007). The fundamental differences between lncRNAs and mRNAs are often embedded within intricate feature patterns that require considering the combined effects of multiple features for clear differentiation. Moreover, univariate methods are sensitive to outliers and noisy data, potentially leading to the selection of irrelevant or misleading feature subsets and adversely impacting classification performance (Bolón‐Canedo *et al*., 2013).

Overall, for large sample datasets, the RFI‐based feature selection strategy is the optimal choice for distinguishing plant lncRNAs from mRNAs, as it balances high predictive performance with computational efficiency.

### Novel mathematical features and their biological significance

A major innovation of PlantLncBoost is the incorporation of novel mathematical features, specifically complex Fourier average and atomic Fourier amplitude. These features capture intricate aspects of lncRNA sequences that are not revealed by traditional sequence‐based descriptors (Messaoudi *et al*., 2014). For instance, the Fourier transform‐based features offer a mathematical perspective on sequence periodicity and frequency‐domain characteristics, which are distinct between lncRNAs and mRNAs due to differences in coding potential (Afreixo *et al*., 2004). An important characteristic of mRNA is its highly regularized coding region composed of triplet codons, which exhibit a clear three‐base periodicity (Tiwari *et al*., 1997). When applying the Fourier transform to mRNA sequences, this periodicity manifests as prominent peaks in the frequency spectrum (Tiwari *et al*., 1997; Nair & Sreenadhan, 2006). By contrast, lncRNA, which does not encode proteins, does not adhere to this three‐base periodicity. As a result, the Fourier spectrum of lncRNA may lack these specific frequency peaks and instead display a more dispersed or complex frequency distribution (Rajesh & Krishnamachari, 2023). The use of such mathematical descriptors ensures that PlantLncBoost is not restricted by the poor sequence conservation that typically impairs other models, thus enhancing its generalization ability across species.

Moreover, the biological relevance of the key features should not be understated. ORF coverage effectively captures the translational potential of RNA sequences, allowing the model to differentiate between coding and noncoding RNAs based on their ability to form complete ORFs, which is generally lacking in lncRNAs (Kong *et al*., 2007). The Fourier‐based features, such as complex Fourier average and atomic Fourier amplitude, reflect inherent sequence periodicity and composition (Tiwari *et al*., 1997), which may be related to the structural or functional motifs critical for lncRNA activity in gene regulation.

### Generalization and reliability in novel lncRNA discovery

The integration of novel features, combined with the use of the CatBoost algorithm, allows PlantLncBoost to significantly improve the accuracy and generalization of lncRNA identification across a broad range of plant species. This is crucial for the discovery of novel lncRNAs, particularly in less studied plant genomes. Unlike existing tools that often exhibit biases or reduced performance on new species, PlantLncBoost demonstrates consistent results in identifying lncRNAs across 20 diverse plant species, including both model organisms and nonmodel plants. The ability of PlantLncBoost to generalize effectively, even to phylogenetically distant species, underscores its reliability and potential utility in discovering new lncRNAs in unexplored plant genomes.

The improvement in generalization directly translates into greater reliability of research outcomes. By reducing the species‐specific biases that have historically impacted the accuracy of lncRNA prediction models, PlantLncBoost provides a robust tool that can be used confidently across different plant species. This is a significant contribution to the field, as it enables researchers to extend the study of lncRNA functions beyond well‐characterized species, facilitating new discoveries in plant biology and offering insights into the evolutionary conservation and diversification of lncRNAs (Palos *et al*., 2023; Traubenik *et al*., 2024).

### Implications and limitations

The success of PlantLncBoost in improving the identification of lncRNAs across a wide variety of plant species has several implications for future research. First, the use of advanced feature selection and gradient boosting models could be further extended to explore other noncoding RNA classes or even protein‐coding genes, particularly in taxa where genomic resources are limited. Additionally, the key features identified in this study, especially those derived from Fourier analysis, could provide new avenues for exploring the structural and regulatory roles of lncRNAs, which remain largely unexplored in many plant species.

However, there are limitations to the current implementation of PlantLncBoost that warrant further exploration. While the model has proven effective in generalizing across diverse species, the reliance on numerical features means that certain biological contexts, such as tissue‐specific expression or epigenetic regulation, are not explicitly modeled. Future enhancements could involve integrating more biological context, such as chromatin accessibility or interaction networks, to further improve the specificity of lncRNA predictions (Ross *et al*., 2021). Additionally, expanding PlantLncBoost to include deep learning approaches that can learn more abstract representations from raw sequence data could further boost its prediction capabilities and adaptability.

### Conclusion

In summary, PlantLncBoost represents a significant advancement in plant lncRNA identification by effectively addressing the challenge of weak generalization that arises from poor sequence conservation. By leveraging novel mathematical features and gradient boosting algorithms, PlantLncBoost achieves high accuracy and generalizability, enabling the reliable study of lncRNAs in a wide range of plant species. The identification of key features, such as ORF coverage and Fourier‐based descriptors, provides deeper insights into the intrinsic properties of lncRNAs, offering a foundation for future studies into their structural and functional roles in plants.

## Competing interests

None declared.

## Author contributions

J‐FM and L‐BJ conceived and designed the study. X‐CT collected the data and conducted the analyses. X‐CT drafted the manuscript, while J‐FM and L‐BJ provided revisions and enhancements. SN performed the comparative analysis of the models. DSD and ARP contributed to validate the results and provided critical suggestions for improvement. All authors reviewed and approved the final version of the manuscript.

## Disclaimer

The New Phytologist Foundation remains neutral with regard to jurisdictional claims in maps and in any institutional affiliations.

## Supporting information


**Fig. S1** Density distributions of lncRNAs and mRNAs on three key features across three plant species.
**Fig. S2** The 10‐fold cross‐validation of PlantLncBoost, LncFinder‐plant, and CPAT‐plant models.
**Fig. S3** Density distributions of lncRNAs and mRNAs on *k*‐mer values in *Arabidopsis thaliana*, *Oryza sativa*, and *Populus trichocarpa*.
**Fig. S4** Density distributions of lncRNAs and mRNAs on Fickett scores values in *Arabidopsis thaliana*, *Oryza sativa*, and *Populus trichocarpa*.

**Table S1** The numbers of lncRNA and mRNA across 20 species in the test dataset.
**Table S2** Comprehensive evaluation of 10 prediction tools for identifying experimentally validated plant lncRNAs across diverse species.
**Table S3** The 1662 features for lncRNA identification model construction.
**Table S4** Fivefold cross‐validation evaluation of CatBoost, XGBoost, and LightGBM algorithms for lncRNA identification.
**Table S5** Time efficiency comparison of CatBoost, XGBoost, and LightGBM algorithms.
**Table S6** Optimal hyperparameter combinations for CatBoost under different feature selection methods.
**Table S7** Five cross‐validation evaluation metrics for CatBoost under different feature selection methods.
**Table S8** Optimal CatBoost hyperparameter combinations for top 20 features selected by random forest importance.
**Table S9** Cross‐validation evaluation metrics for CatBoost with top 20 features selected by random forest importance.
**Table S10** Performance of 10 lncRNA identification models in terms of sensitivity, specificity, accuracy, precision, F1‐score, and AUC across 20 plant species.
**Table S11** Performance of PlantLncBoost in terms of sensitivity, specificity, accuracy, precision, F1‐score, and AUC on the average level across 20 plant species.
Please note: Wiley is not responsible for the content or functionality of any Supporting Information supplied by the authors. Any queries (other than missing material) should be directed to the *New Phytologist* Central Office.

## Data Availability

The PlantLncBoost tool is freely accessible on GitHub at https://github.com/xuechantian/PlantLncBoost. The training dataset used in this study can be found within the PlantLncBoost repository at https://github.com/xuechantian/PlantLncBoost/tree/master/data. The test dataset is available at https://github.com/xuechantian/PlantLncBoost/tree/master/data/testdata. The collected experimentally validated lncRNAs are available at https://github.com/xuechantian/PlantLncBoost/tree/master/data/validated_lncRNA.fasta. The comprehensive lncRNA analysis pipeline is accessible at https://github.com/xuechantian/Plant‐LncRNA‐pipeline‐v2.
