# Supplementary material for: PlantLncBoost: key features for plant lncRNA identification and significant improvement in accuracy and generalization
Source: New Phytol. 2025 May 27;247(3):1538–49. doi: 10.1111/nph.70211 (PMC12222927; doi:10.1111/nph.70211)
Supplement: Supplementary file 1 — Fig. S1 Density distributions of lncRNAs and mRNAs on three key features across three plant species. Fig. S2 The 10‐fold cross‐validation of PlantLncBoost, LncFinder‐plant, and CPAT‐plant models. Fig. S3 Density distributions of lncRNAs and mRNAs on k‐mer values in Arabidopsis thaliana, Oryza sativa, and Populus trichocarpa. Fig. S4 Density distributions of lncRNAs and mRNAs on Fickett scores values in Arabidopsis thaliana, Oryza sativa, and Populus trichocarpa. [file NPH-247-1538-s001.pdf]

New Phytologist Supporting Information

Article title: PlantLncBoost: key features for plant lncRNA identification and significant improvement in accuracy and generalization

Authors: Xue-Chan Tian, Shuai Nie, Douglas Silva Domingues, Alexandre Rossi Paschoal, Li-Bo Jiang, Jian-Feng Mao

Article acceptance date: 15 April 2025

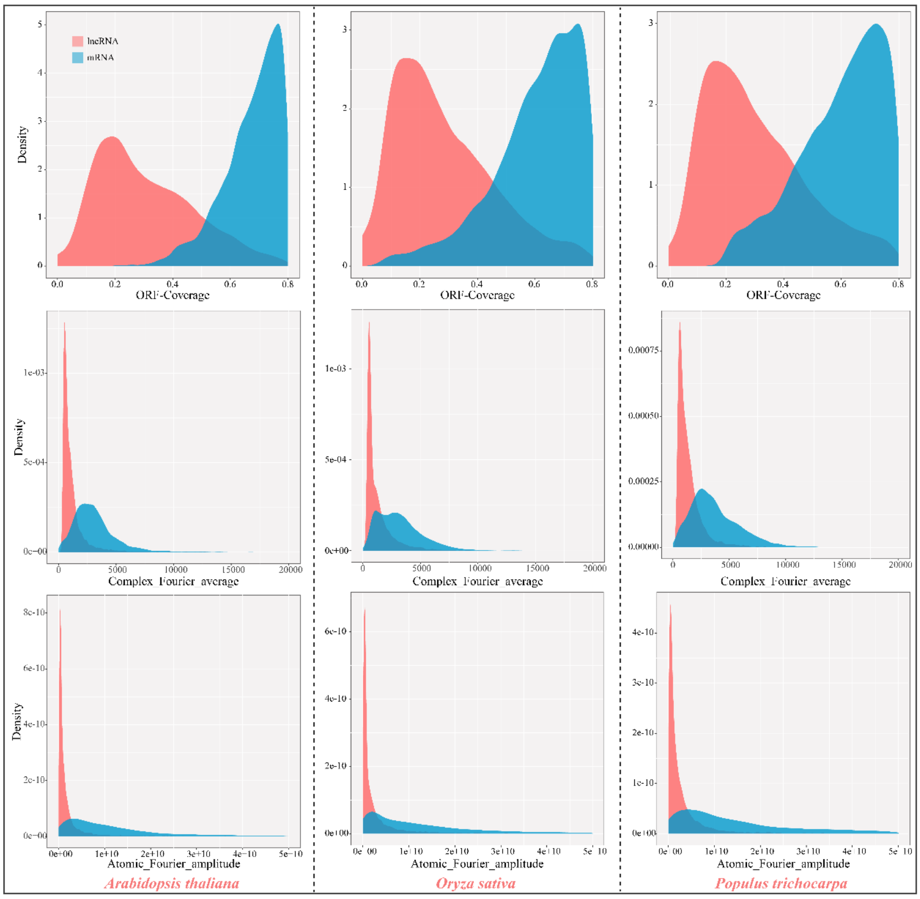

Fig. S1. Density distributions of lncRNAs and mRNAs on three key features across three plant species.

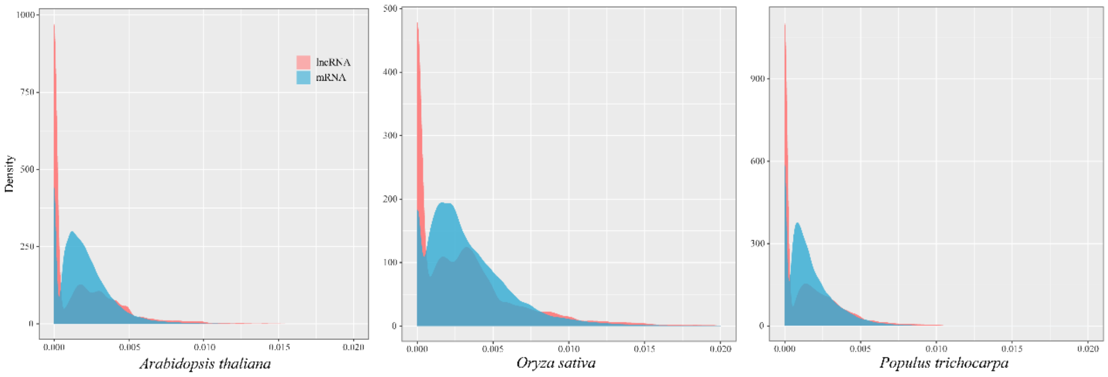

Fig. S2. Density distributions of lncRNAs and mRNAs on *K*-mer values in *A. thaliana*, *O. sativa*, and *P. trichocarpa.*

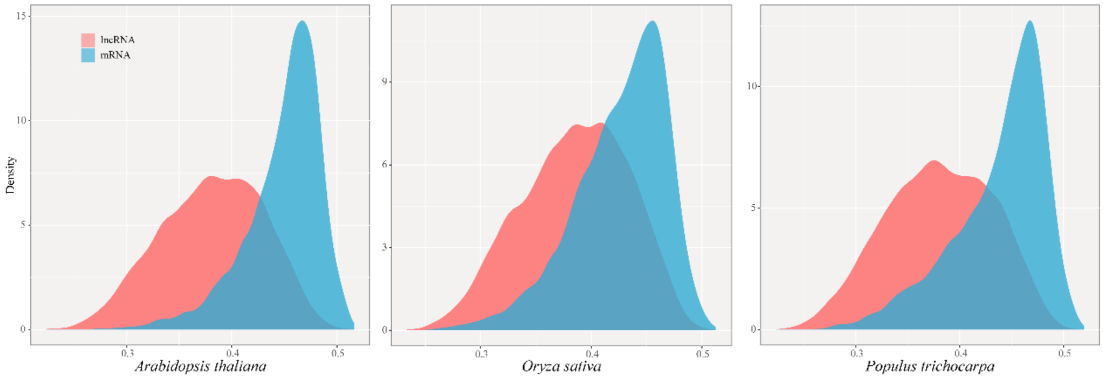

Fig. S3. Density distributions of lncRNAs and mRNAs on Fickett scores values in *A. thaliana*, *O. sativa*, and *P. trichocarpa*.

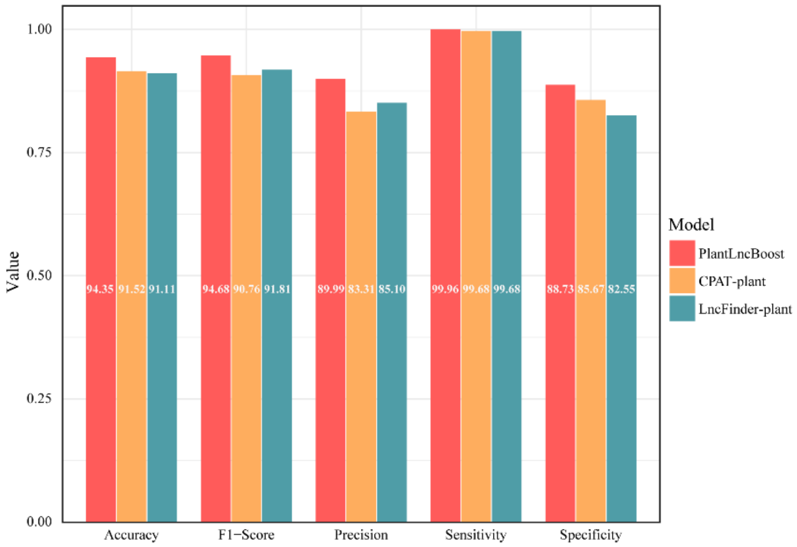

Fig. S4. The 10-fold cross-validation of PlantLncBoost, LncFinder-plant and CPAT-plant model.
